# Non-Invasive Technology That Improves Cardiac Function after Experimental Myocardial Infarction: Whole Body Periodic Acceleration (pGz)

**DOI:** 10.1371/journal.pone.0121069

**Published:** 2015-03-25

**Authors:** Arkady Uryash, Jorge Bassuk, Paul Kurlansky, Francisco Altamirano, Jose R. Lopez, Jose A. Adams

**Affiliations:** 1 Division of Neonatology, Mount Sinai Medical Center, Miami Beach, FL, United States of America; 2 Columbia Heart Source, Columbia University College of Physicians and Surgeons, New York, NY, United States of America; 3 Departments of Molecular Bioscience, School of Veterinary Medicine, University of California Davis, Davis, CA, United States of America; Yale University School of Medicine, UNITED STATES

## Abstract

Myocardial infarction (MI) may produce significant inflammatory changes and adverse ventricular remodeling leading to heart failure and premature death. Pharmacologic, stem cell transplantation, and exercise have not halted the inexorable rise in the prevalence and great economic costs of heart failure despite extensive investigations of such treatments. New therapeutic modalities are needed. *Whole Body Periodic Acceleration* (pGz) is a non-invasive technology that increases pulsatile shear stress to the endothelium thereby producing several beneficial cardiovascular effects as demonstrated in animal models, normal humans and patients with heart disease. pGz upregulates endothelial derived nitric oxide synthase (eNOS) and its phosphorylation (p-eNOS) to improve myocardial function in models of myocardial stunning and preconditioning. Here we test whether pGz applied chronically after focal myocardial infarction in rats improves functional outcomes from MI. Focal MI was produced by left coronary artery ligation. One day after ligation animals were randomized to receive daily treatments of pGz for four weeks (MI-pGz) or serve as controls (MI-CONT), with an additional group as non-infarction controls (Sham). Echocardiograms and invasive pressure volume loop analysis were carried out. Infarct transmurality, myocardial fibrosis, and markers of inflammatory and anti-inflammatory cytokines were determined along with protein analysis of eNOS, p-eNOS and inducible nitric oxide synthase (iNOS).At four weeks, survival was 80% in MI-pGz vs 50% in MI-CONT (p< 0.01). Ejection fraction and fractional shortening and invasive pressure volume relation indices of afterload and contractility were significantly better in MI-pGz. The latter where associated with decreased infarct transmurality and decreased fibrosis along with increased eNOS, p-eNOS. Additionally, MI-pGz had significantly lower levels of iNOS, inflammatory cytokines (IL-6, TNF-α), and higher level of anti-inflammatory cytokine (IL-10). pGz improved survival and contractile performance, associated with improved myocardial remodeling. pGz may serve as a simple, safe, non-invasive therapeutic modality to improve myocardial function after MI.

## Introduction

Myocardial Infarction (MI) and its associated functional derangements may lead to heart failure that affects 2–3% of the population in industrialized countries with a marked rise in those aged >65yr. A combined estimate of ~ 20 million people suffer from heart failure in Europe and the US and its prevalence is increasing. In the US alone direct medical costs approximate $25 billion dollars per year and expected to triple by 2030 [1–4].

Attempts to ameliorate myocardial dysfunction after MI, using pharmacologic, stem cell transplantation, and exercise have been widely explored in experimental and in clinical trials. The search for simple inexpensive therapeutic interventions continues. Whole Body Periodic Acceleration (pGz) is the repetitive, sinusoidal head-foot motion of the body provided by a motion platform. As the body accelerates and decelerates, low amplitude pulses are added to the circulation as a function of platform frequency thereby increasing pulsatile shear stress to the endothelium [5–8, 9]. pGz through its increase of pulsatile shear stress upregulates eNOS and increases its phosphorylation p-eNOS ^(Ser 1177)^ [10] eNOS phosphorylation after pGz is responsible for shear stress dependent vasodilatation and subsequent increase nitric oxide (eNO) release into the circulation [9, 11–13]. pGz applied in a swine model of myocardial stunning induced by whole body ischemia reperfusion injury, acutely improves myocardial function due to increase eNO as well as other cardioprotective mediators [6, 8, 10, 14]. The purpose of this investigation was to test whether pGz applied chronically after focal myocardial infarction in rats would improve functional outcomes from MI.

## Materials and Methods

### Animals and Experimental Design

**Fig 1:**
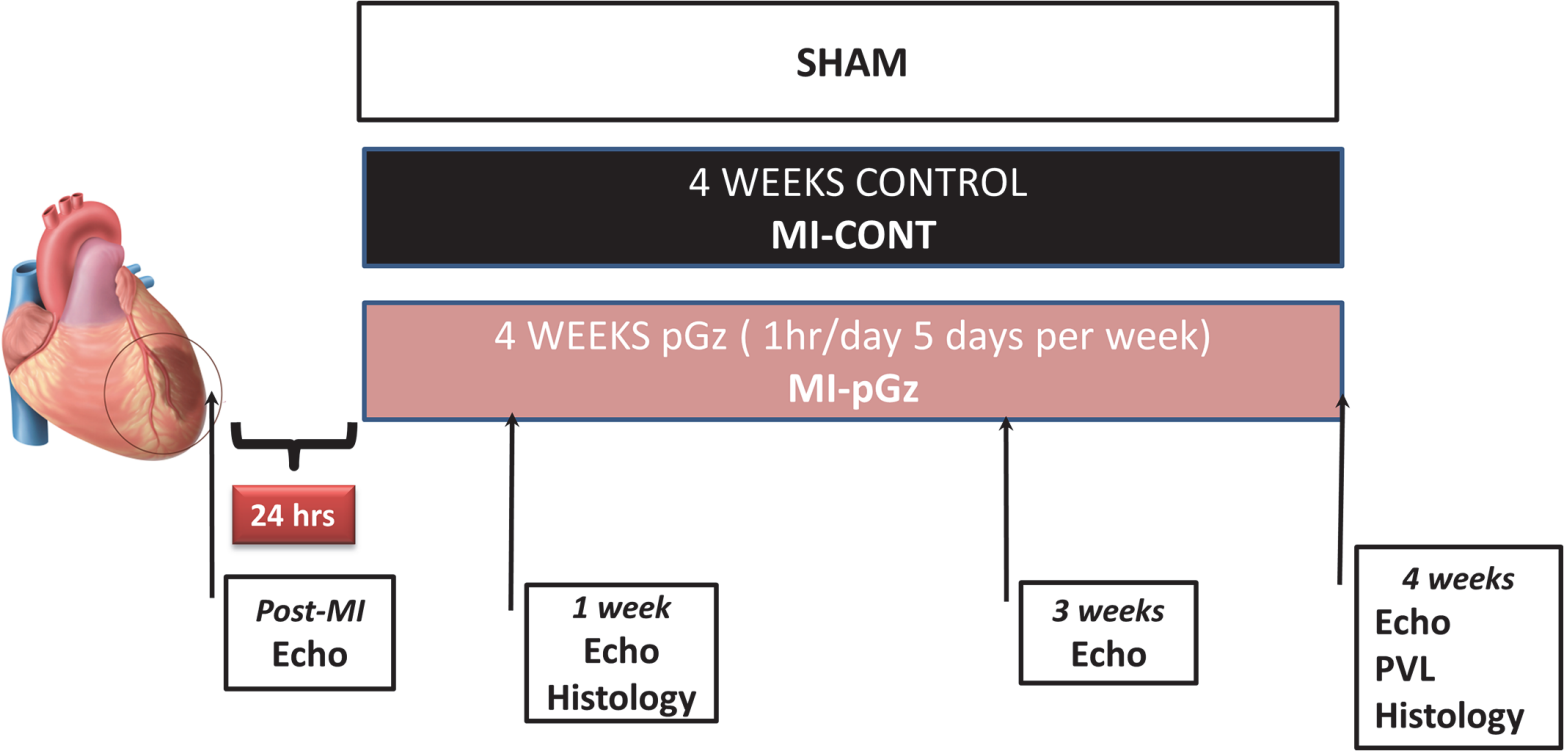
Myocardial Infarction Protocol Schematic. Permanent ligation of left coronary artery without reperfusion was done. pGz or Control were begun 24 hr after MI, and continued for four weeks. Post-MI echocardiograms (Echo) were done immediately post MI, at 1,3,4 weeks. Invasive Pressure Volume Loops were collected at end of study.

The experimental protocol No. 13-08-A-03 was approved by the Mount Sinai Medical Center Animal Care and Use Committee and conforms to the Guide for the Care and Use of Laboratory Animals published by the National Institutes of Health [NIH Publication No. 85-23, revised 1996]. Adult male Sprague-Dawley rats (300–350 g) were randomly assigned to (*n* = 60): 1) pGz started 24 hr after MI, 1 hr per day 5 days per week for four weeks (MI-pGz) (n = 20); 2) A control group (MI-CONT) animals were placed on the pGz platform starting at 24 hr after MI but the platform was not actuated (n = 20); 3) Sham group, animals where anesthetized but no MI was induced (Sham) (n = 10) (Fig. 1).

### pGz Protocol

After experimental MI was induced by permanent ligation of the left coronary artery, the animals recovered for 24 hr. Awake animals were placed in a restraint for 1hr/day 5 days per week for four weeks. Optimal pGz frequency of 360 cpm and peak acceleration amplitude of Gz ± 2.9 m/sec^2^ in rats for increased release of eNO was utilized [10, 13]. pGz was administered at the same time of the day for the various study groups. Animals who did not receive pGz were placed in the same restraint for the same number of days as the experimental groups.

### Myocardial Infarction Model

Animals were anesthetized with Ketamine/Xylazine (80/10 mg/kg IM) intubated and ventilated with O_2_ using a small-animal ventilator (Kent Scientific Corporation, Torrington, CT). Body temperature was maintained at 37.5 ± 0.5°C with a heated operating platform. Cardiac electrical activity was monitored continuously with 3 lead electrocardiogram. The tail vein was catheterized for intravenous fluids and drug administration. Left thoracotomy was performed and a permanent ligature placed around left anterior coronary artery at the mid-level. The thorax was closed in layers with silk sutures. Animals were extubated and recovered in their cages maintaining a thermo-neutral environment and received analgesia [Buprenex (buprenorphine) 0.1 mg/kg] immediately after surgery and every 6 to 12 hr. based on pain assessment. Animals are monitored hourly or until upright and ambulating. Once ambulating animals are assessed for post procedural pain every 4 hr. for the initial 48 hr., thereafter once per day until completion of the protocol. A modified score sheet system based on post procedural pain assessment of behavior, physiological stress signs and respirations is used to tabulate assessments (S1 Table) [15–17].

### Echocardiogram and Invasive Hemodynamic Measurements

Echocardiograms where performed post MI at 1, 3,and 4 weeks of pGz or Control using an HP Sonos 2000 ultrasound imaging system (Hewlett-Packard, Andover, MA USA) with a 7.5 mHz transthoracic transducer. 2D/M-Mode techniques were applied to assess functional changes. Echocardiograms were performed in long- and short-axis following myocardial infarction in close-chest sedated rats. The chest was shaved and covered with ultra sound gel in order to provide air-free insonation. The left ventricle (LV) was visualized by middle long and short axis images. Middle long and short axis views were taken from the right ventricular side in order to depict the left ventricle at a lower sector-angle. By reducing insonation angle without reducing spatial resolution, the technique provided images with a 76 frame rate. Three cardiac cycles were acquired and averaged by software. The correct tracking of border zones was visually controlled and manually corrected. Ejection time was defined by Doppler-registered aortic valve opening and closure. Conventional measurements (LV diameters, anterior wall (AW) and posterior wall (PW) thickness and thickening) were obtained from grayscale M-mode tracings. LV end-systolic and end-diastolic volumes and LV ejection fraction (LVEF) were measured by Simpson’s method from two-dimensional parasternal long- and short-axis views [18–20].

After four weeks of pGz or Control the animals where anesthetized and catheterization was performed with a Millar Catheter SPR-869 (Millar Instruments, Houston TX, USA). The conductance catheter was calibrated by a cuvette calibration method using an actual blood sample in cuvette between 50 and 300 μl. In vivo the conductance signal was calibrated using hypertonic saline. An intravenous bolus of 50 μl of 20% saline was used to perform calibration. In order to decrease preload, a small abdominal incision was performed to localize and perform inferior vena cava (IVC) occlusions. PVL where continuously recorded at baseline, after saline infusion, and during and after IVC occlusions. Recording and calculations were performed using data acquisition software (LabChart7Pro, ADInstruments, Colorado Springs, CO).

### Sham Surgery

Sham protocol was identical to MI experiments with the exception of coronary artery ligation. At the completion of the sham surgery, animals were recovered and allowed to remain in their cage with free access to chow and water for the remainder of their study time period.

Animals which met pre-established humane endpoints for euthanasia or completed the experimental protocol were euthanized by a dose of Ketamine 90mg/kg and Xylazine 25mg/kg, followed by pentobarbital 100mg/kg IP, until absence of corneal and pedal reflex, and no electrical activity on ECG, and decapitation via guillotine, a method approved by the American Veterinary Medical Association Guidelines on Euthanasia. Perfusion and or organ harvesting was performed as per protocol [21–24].

### Detection and Assessment of Infarcted Area, Transmurality and Fibrosis

Validation of coronary occlusion was performed by our laboratory according to the procedure previously described [25]. In a separate cohort of animals (n = 10) infarct size was determined after 24 hr. of coronary occlusion to determine infarct size. At the end of the study (four weeks after ligation) and after all hemodynamic measurements, the aorta was clamped and the hearts were perfused with 10mL of saline through a cannula in the ascending aorta to wash out the blood from the myocardium [26]. After saline perfusion, Evans Blue (EB) was injected into the ascending aorta to separate the non-at-risk area from the risk area. The hearts were cut out and cut in 3, 3mm segments from apex to base parallel to the atrioventricular groove. The segments were incubated for 30 minutes in 2,3,5-triphenyltetrazolium chloride (TTC) at 37°C in the dark. The segments were fixed between two glass sheets and non-at-risk area, the area-at-risk and the necrotic area were determined by planimetry [27]. The basal side of the segments was measured to better distinguish between myocardium stained by EB and TTC. Segments for comparison were chosen on the basis of reproducibility of area-at-risk to perfused myocardium ratio between animals. Images of the segments were taken with a digital camera set to 60 x magnifications through a dissecting microscope (Olympus, Center Valley, PA). Viable myocardium (TTC red stained) and infarcted (TTC unstained) areas non-at-risk area (EB–blue stained) were measured using a computer program (Adobe Photoshop, Adobe Systems Incorporated, San Jose, CA). The percentage share of each the preceding areas was calculated.

Area at risk measured by the Evans Blue (EB) perfusion-staining and expressed as percent of whole heart. Necrosis was measured by TTC staining and expressed as percent of each myocardial segment. To determine transmurality of the infarct scanned images of the segments were geometrically divided into a 6-sector model using the anterior and inferior insertion of the right ventricle to the left ventricle as markers [28]. Apical, middle and basal necrosis was defined. The sectors were divided into the following groups on the background of the distribution of necrotic myocardium: transmural necrotic (necrosis>50%), subendocardial necrotic (1–50% necrosis) and viable (necrosis = 0). The combination of the two latter groups is referred to as predominantly viable (0–50%).

Transmural necrotic sectors display thinning of the myocardial circumferential areas of left ventricular wall. Transmurality of the infarct was defined as the sum of the epicardial and endocardial infarct circumference divided by the sum of the total LV epicardial and endocardial circumferences using computer-based planimetry [29]. Fibrosis was measured by Masson's Trichrome (MT) staining and expressed as percent of left ventricle in a segment. Hearts were washed three times in PBS at 4°C. Hearts were then cut into three transverse segments. Each segment was fixed in 10% para-formaldehyde and embedded in paraffin. The middle transverse segment was sectioned and stained with MT for both wall thickness (epi-to-endocardial distance) and fibrosis (blue stained collagen fibers) measurements. The mean left ventricle (LV) wall thickness, fibrosis and total LV area were measured from three equidistant points.

### Tissue Preparation for detection of eNOS, p-eNOS, iNOS TNF-α, IL-6 and IL-10

To assess the effects of pGz on eNOS, p-eNOS and iNOS and inflammatory TNF-α, IL-6 and anti-inflammatory cytokine IL-10 protein levels were determined. Samples of the left ventricle were removed and immediately frozen in liquid nitrogen for Western Blot and cytokine protein chemiluminescent analysis. Care was taken to consistently harvest the same region of the left ventricle from all animals. Sections of the left ventricular myocardium were individually minced and homogenized at 4° C followed by one step total protein extraction with an Extraction Buffer System (Invitrogen Corporation, Carlsbad, CA) according to the manufacturer’s protocol. Homogenates were centrifuged at 4°C for 20 minutes at 12,000 rpm. The supernatant was decanted and assayed for total protein content. The extracted total cardiac tissue protein was measured by the BCA Protein Assay (Thermo Fisher Scientific, Waltham, MA) on a SpectraMax Plate Reader (Molecular Devices, Sunnyvale, CA). Individual proteins were then analyzed by Western Blot. Equal amounts of total protein were separated on 4–12% NuPAGE Novex Bis-Tris SDS-PAGE Gels (Invitrogen Corporation, Carlsbad, CA) and transferred to Immobilon-FL PVDF membrane (Millipore Corporation, Billlerica, MA). The PVDF membrane was treated with a blocking agent (GE Healthcare Bio-Sciences Corporation, Piscataway, NJ) and probed with primary, fluorescein-linked secondary antibodies as well as anti-fluorescein alkaline phosphatase conjugate. Primary antibodies to eNOS, p-eNOS^(Ser1177)^ iNOS, and GADPH (Glyceraldehyde-3-phosphate dehydrogenase) as an individual protein loading control (Santa Cruz Biotechnology, Inc. Santa Cruz, CA.) were employed. TNF-α, IL-6, IL-10 where determined using Rat Cytokine Kit (R&D Systems Inc., Minneapolis, MN). Equal amounts of protein 400 μg were used for analysis. Cytokine levels where normalized to Sham samples. Blots were visualized by Enhanced Chemifluorescence (ECF) (GE Healthcare Bio-Sciences Corporation, Piscataway, NJ) on Storm 860 Imaging System (GE Healthcare Bio-Sciences Corporation, Piscataway, NJ). The Storm 860 Imaging System exhibits a linear response to fluorescent signal intensities and protein levels were quantified using ImageQuant software (GE Healthcare Bio-Sciences Corporation, Piscataway, NJ).

### Data Analysis

Primary outcomes are survival and myocardial infarct size. Secondary outcomes are myocardial fibrosis, hemodynamics and protein expression. Myocardial contractility measurements were analyzed using a two-way analysis of variance with repeated measures. When appropriate, post hoc Fisher’s least significant difference test was carried out. Myocardial protein analysis parameters were subjected to analysis of variance with a Fisher’s least significant difference test used *post hoc*. Statistical significance was established at p*<*0.05. Sample size was calculated using Statistica (StatSoft, Tulsa, OK) based on power analysis with α = 0.05 and power 0.80. All data are expressed as Mean ± SD.

## Results

### Survival and Myocardial Infarct Size and Myocardial Fibrosis

**Fig 2:**
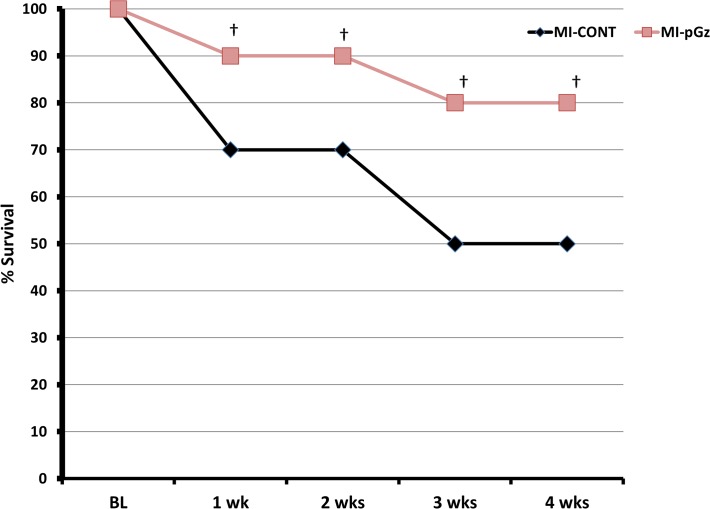
pGz Improves Survival After Myocardial Infarction. *Kaplan-Meir Survival Curves*. Survival during the four weeks after myocardial infarction in MI-pGz and MI-CONT groups. † p< 0.01.

All animals survived myocardial infarction without reperfusion at 24 hr. At 1 week post MI, 70% of the MI-CONT and 90% MI-pGz animals survived (p< 0.01). Four weeks after MI, 80% of the pGz treated animals survived and 50% of the MI-CONT (p< 0.001) (Fig. 2).

**Fig 3:**
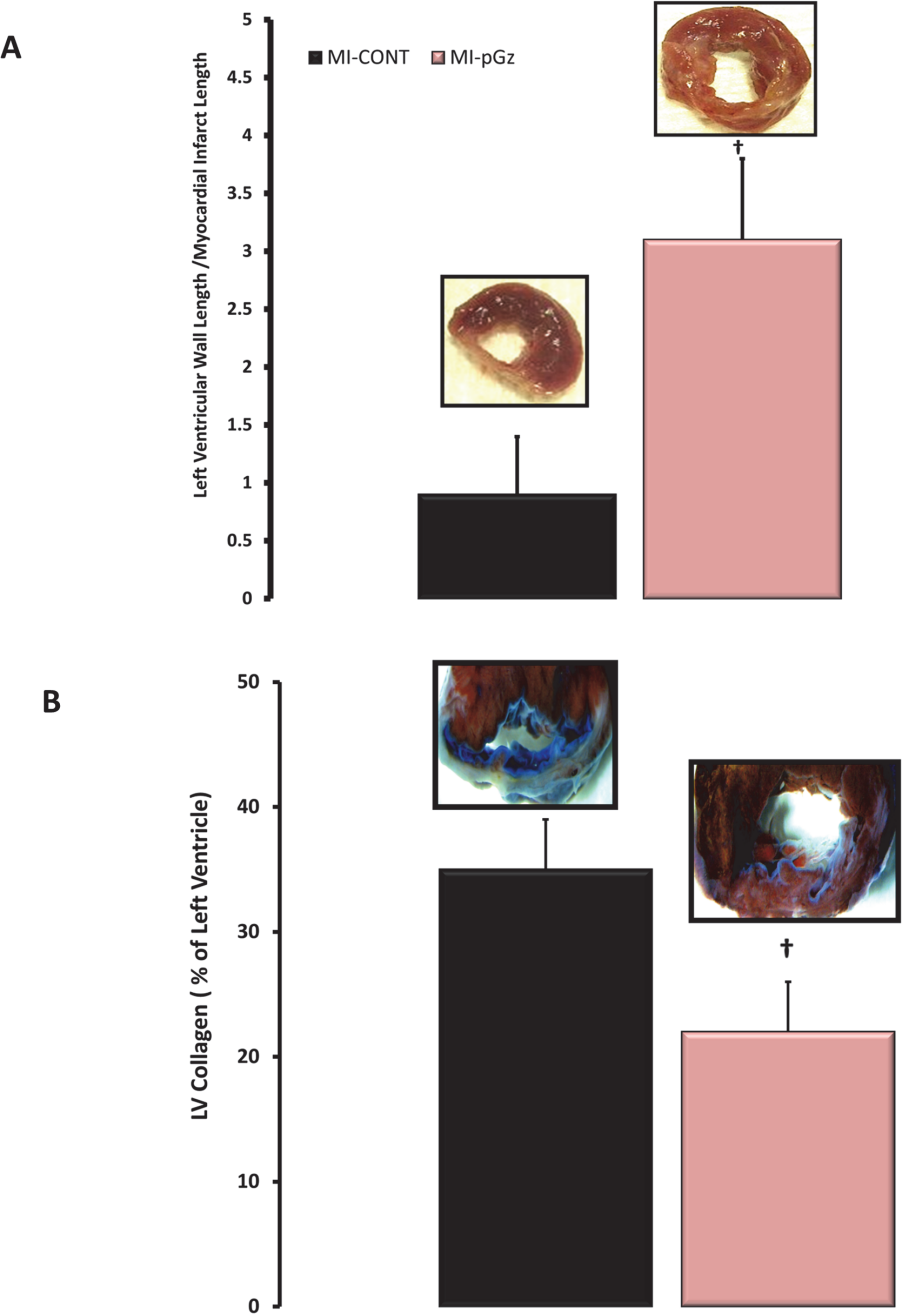
pGz Reduced Infarct Size, Transmurality and Fibrosis. A. The ratio of left ventricular wall to myocardial infarct length in MI-CONT and MI-pGz, with representative microscopic findings. †p< 0.01 MI-CONT vs. MI-pGz. B. Left Ventricular Collagen as a % of the left ventricle in MI-CONT and MI-pGz, with representative microscopic findings. Blue staining denotes fibrosis †p< 0.01 MI-CONT vs. MI-pGz.

Infarct size prior to randomization was 38±4%. Treatment with pGz for four weeks decreased transmurality of the infarct from 60± 5% for MI-CONT to 48±4% for MI-pGz (p< 0.01). The ratio of left ventricular wall thickness to the MI thickness was significantly smaller in MI-CONT 0.9±0.5 compared to 3.1 ± 0.7 for MI-pGz (p< 0.01), additionally the amount of left ventricular collagen was 35±4% in MI-CONT compared to 22±4% in pGz treated animals (p< 0.01)(Fig. 3).

### Hemodynamics and Myocardial Performance

**Fig 4:**
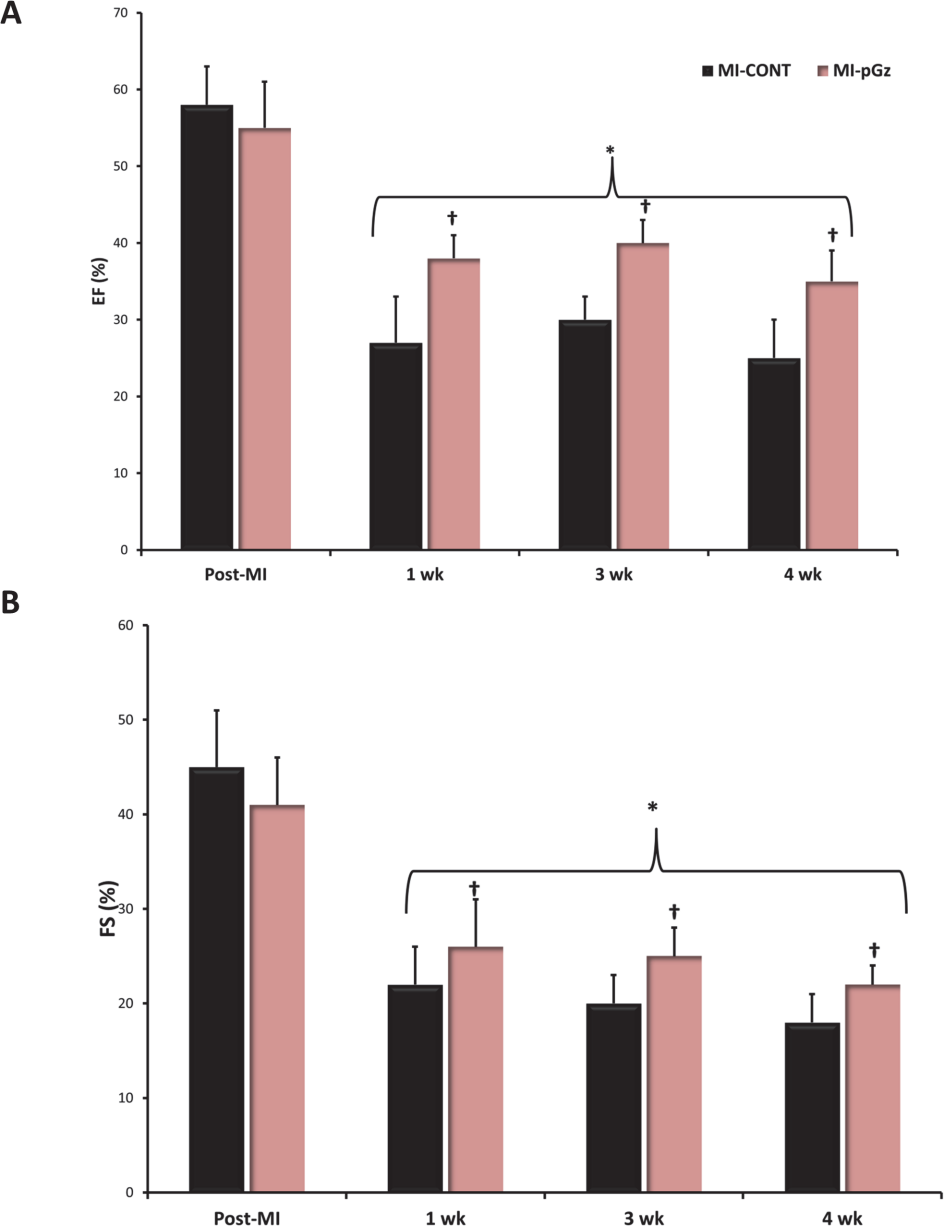
Ejection Fraction and Fractional Shortening. A. Ejection Fraction (EF %) determined by echocardiogram immediately after MI (Post-MI) and at 1,3,4 weeks. * p< 0.01 MI-CONT and MI-pGz at 1,3,4 weeks vs. Post-MI. †p< 0.01 MI-CONT vs. MI-pGz. B. Fractional Shortening (FS%) determined by echocardiogram immediately after MI(Post-MI) and at 1,3,4 weeks. * p< 0.01 MI-CONT and MI-pGz at 1,3,4 weeks vs. Post-MI. †p< 0.01 MI-CONT vs. MI-pGz.

Ejection fraction and Fractional shortening significantly decreased at 1, 3, and four weeks after MI in both MI-CONT and MI-pGz compared to the immediate post MI time period. Over the four week post MI period, EF decreased an average of 35% from immediate post MI values in MI-pGz, while in MI-CONT the average decrease was 53%. Similarly FS, decreased on average 46% in MI-pGz, and 56% in MI-CONT (p< 0.01) (Fig. 4).

**Fig 5:**
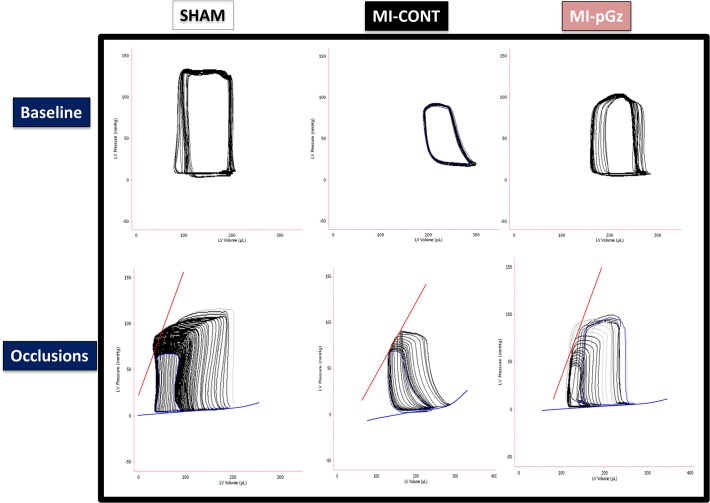
Invasive Pressure Volume Relationship. Representative loops for the pressure volume relationship of the left ventricle four weeks after myocardial infarction determined at baseline, and after reduction of preload via occlusion in Sham, MI-CONT and MI-pGz.

As expected and in agreement with other studies, MI significantly decreased invasive measures of afterload, preload, integrated myocardial performance, and contractility [30, 31]. Four weeks of pGz significantly improved left ventricular end systolic pressure (LVESP) and invasive contractility measures, including; dp/dt, end systolic and end diastolic pressure volume relation (ESPVR, EDPVR), and significantly improved LVEDP, compared to MI-CONT. (Table 1, Fig. 5).

**Table 1:** Invasive Hemodynamic Measures of Myocardial Function.

	**Sham**	**MI-CONT**	**MI-pGz**
Heart Rate *(bpm)*	322(15)	348(20)	351(11)
***Integrated Performance***			
EF(*%*)	67(1)	35(3.4)*	48(1.3)* †
SW *(mmHgx*μ*l)*	9959(288)	8342(472)*	8478(380)*
***Afterload***			
LVESP*(mmHg)*	149(3)	133(2.5) *	140(1.4)* †
***Preload***			
LVEDP*(mmHg)*	9(1)	22(6)*	11(3)†
***Contractility***			
dP/dtmax (mmHg/s)	13,593(228)	7,742(225)*	11,916(14) * †
ESPVR *(mmHgx*μ*l)*	2.64 (0.19)	1.08(0.06)*	1.58(0.16) * †
EDPVR *(mmHgx*μ*l)*	0.041(0.003)	0.083(0.004)*	0.063(0.011) * †

Invasive hemodynamic measures obtained from the analysis if the pressure volume relationship of myocardial function at four weeks after myocardial infarction in Sham, MI-CONT and MI-pGz. Ejection Fraction (EF%), Stroke work (SW), Left Ventricular End Systolic Pressure (LVESP), Left Ventricular End Diastolic Pressure (LVEDP), End Systolic Pressure Volume Relationship (ESPVR), and End Diastolic Pressure Volume Relationship (EDPVR).

* p< 0.01 MI-CONT and MI-pGz vs. Sham.

†p< 0.01 MI-CONT vs. MI-pGz.

### Protein expression of eNOS, p-eNOS, iNOS, TNF-α, IL-6 and IL-10

**Fig 6:**
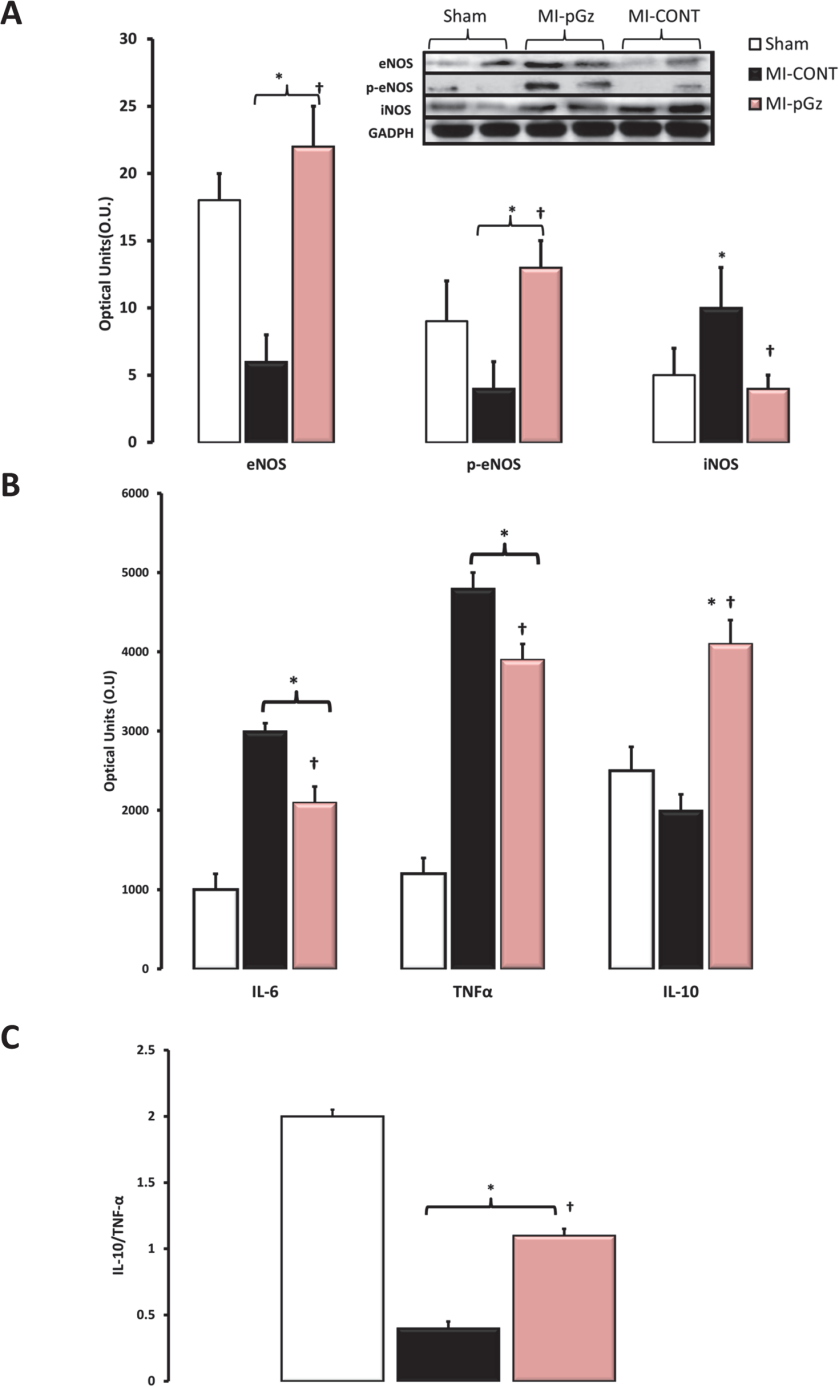
pGz Increases eNOS Signaling and IL-10 Expression, whereas it Reduces iNOS, TNF-α and IL-6 Expression. A. Left ventricular protein content of endothelial derived nitric oxide synthase (eNOS) and phosphorylated eNOS (p-eNOS) as well as inducible nitric oxide synthase (iNOS), for Sham, MI-CONT and MI-pGz with representative immune blots for each group.* p< 0.01 MI-CONT and MI-pGz vs. Sham. †p< 0.01 MI-CONT vs. MI-pGz. B. Left ventricular protein content of the pro-inflammatory cytokines; Interleukin 6 (IL-6) and Tumor necrosis factor alpha (TNF-α) and the anti-inflammatory cytokine; Interleukin 10 (IL-10) in Sham, MI-CONT and MI-pGz. * p< 0.01 MI-CONT and MI-pGz vs. Sham †p< 0.01 MI-CONT vs. MI-pGz. C. Ratio of anti-inflammatory IL-10 to pro-inflammatory TNF-α cytokines. * p< 0.01 MI-CONT and MI-pGz vs. Sham †p< 0.01 MI-CONT vs. MI-pGz.

Compared to sham animals four weeks after myocardial infarction, there was a significant decrease in left ventricular protein content of eNOS (66%), p-eNOS(55%), and a significant increase in iNOS (100%). pGz after MI significantly increased eNOS (78%), p-eNOS (56%) and abrogated the increase in iNOS to only 20% compared with MI-CONT (Fig. 6).

pGz after MI decreased left ventricular protein content of IL-6 and TNF-α to 33% and 25% respectively of MI-CONT (p< 0.01). pGz also significantly increased the anti-inflammatory signaling cytokine IL-10, 100% of MI-CONT (p< 0.01). Additionally, the ratio of IL-10/ TNF-α was also significantly increased 1.1± 0.05 vs. 0.4±0.05 in MI-pGz vs. MI-CONT (p< 0.01) (Fig. 6.).

## Discussion

Our study assessed the beneficial functional effects of treatment with pGz after experimental MI in rats. pGz administered one hour daily significantly improved survival and myocardial function. It decreased myocardial fibrosis and transmurality of the infarct, associated with increased eNOS, p-eNOS, and IL-10 along with decreased expression of TNF-α, IL-6 and iNOS.

### pGz Improves Survival and Hemodynamics

pGz improved survival and contractile performance in this post MI model of heart failure. pGz has been shown to increase myocardial p-eNOS and NO and induce genomic up regulation of eNOS. The present study shows that daily pGz after MI significantly increases eNOS and p-eNOS, while significantly decreasing iNOS expression. Additionally pGz has also been shown to increase myocardial nNOS [10, 13, 32, 33]. The role of NO in cardioprotection and as a therapeutic modality in myocardial repair and remodeling, has been extensively reviewed by other investigators with both eNOS and nNOS playing a significant role in lessening injury, reducing apoptosis, decreasing myocardial fibrosis and ameliorating injury mediated by both reactive oxygen (ROS) and nitrogen oxide species (RNS) [34, 35]. Pharmacologic enhancement of eNOS and nNOS has also been shown to be cardioprotective after MI in mice [36] and enhancing mesenchymal cell therapy in swine [37]. The role of iNOS in myocardial repair and remodeling is less clear and controversial; with studies suggesting that increased NO production thru overexpression of iNOS contributes to detrimental cardiac remodeling in heart failure, [38–40] and lack of iNOS improving cardiac reserve post MI [41]. In contrast others showed a protective role for iNOS in LV remodeling in a cardiomyopathy hamster heart model [42]. Recently, potentiation of NO signaling through inhibition of phosphodiesterase 5 (PDE5) has shown effectiveness in preventing cardiac hypertrophy and heart failure induced by pressure overload in animal models as well as, promising for human heart failure, diabetic cardiomyopathy, and pulmonary hypertension [43].

### pGz Treatment Decreases Myocardial Fibrosis

Daily pGz treatment commencing 24 hr. after ischemia for four weeks improved the amount of viable left ventricle as evidenced by a higher ratio of left ventricular wall thickness to myocardial infarct thickness, and lower transmural extension of the infarct. Additionally, left ventricular collagen content, a measure of myocardial fibrosis, was reduced by 37% in pGz treated animals. The latter was associated with a decrease in pro-inflammatory cytokines, and increase in anti-inflammatory signaling cytokine.

Cytokines and chemokines have been shown to be important signaling molecules for early fibrosis [44–49]. In our model of MI, TNF-α and IL-6 measures of pro-inflammation were reduced in pGz treated animals while IL-10 an anti -inflammatory signaling cytokine was increased in pGz treated animals. Additionally, the balance of IL-10/ TNF- α in pGz treated animals was shifted to the more favorable anti-inflammatory ratio. Other investigators have also shown that both exercise and statins may also shift this ratio [50, 51].

During myocardial ischemia, TNF- is released from macrophages, monocytes and mast cells and after reperfusion TNF- is expressed and secreted in both cardiomyocytes and fibroblasts[52]. TNF- has an ambivalent role in MI depending on the receptor subtype that is activated. Cell signaling through TNFR1 exacerbates remodeling, hypertrophy and apoptosis in heart failure, whereas TNFR2 has opposite effects [53].

Physical exercise decreases symptoms of coronary heart disease and is at the core of cardiac rehabilitation [54, 55]. Exercise as a method ameliorating ventricular remodeling after MI has been reported in various animal models including rats [50, 56], and humans [57, 58]. In rats and pigs, we previously showed that a single hour of pGz induces upregulation and activation of nNOS, eNOS and Akt and their respective phosphorylated proteins in the heart within 4 hr. of exposure [8, 10]. In the current study, pGz after MI also increased eNOS protein expression and phosphorylation.

Taken together our findings suggest that pGz post MI improves myocardial function, decreases inflammatory signaling, and modifies myocardial remodeling. It is plausible that these changes are at least in part mediated via pGz-induced eNOS up regulation and modulation of an inflammatory phenotype [35, 59–61].

### How Does pGz Improve Survival and Contractile Performance

Several lines of evidence suggest that intracellular Ca^2+^ ([Ca^2+^]_i_) and Na^+^ ([Na^+^]_i_) overload are prime causes of myocardial injury and death during MI [62, 63]. An excessive increase of intracellular Ca^2+^ causes mitochondrial dysfunction, reduces adenosine triphosphate (ATP) production [64, 65], and activates Ca^2+^-dependent protease [66], all of which contribute to impairment of the [Ca^2+^]_i_ homeostasis. In addition, there is an important interplay between [Na^+^]_i_ and Ca^2+^ handling, so that altered levels of [Na^+^]_i_ and Na^+^ transporters can have profound effects on both contractile function and arrhythmogenesis [67, 68]. An increase in [Na^+^]_i_ activates the Na^+^/Ca^2+^ exchanger in its reverse mode (Ca^2+^ in/Na^+^ out) which in turn further elevates [Ca^2+^]_i_ [69] whereas activation of Na^+^/H^+^ exchanger induces acidification of intracellular medium [70]. Thus, both [Ca]_i_ and intracellular pH (pH_i_) in cardiac myocytes depend strongly on [Na]_i_.

Using an *in vitro* model of hypoxia and reoxygenation, we showed that pGz partially prevented intracellular Ca^2+^ and Na^+^ overload and acidification of the intracellular medium during hypoxia and reoxygenation [71]. This favorable effect regarding [Ca^2+^]_i_ and [Na^+^]_i_ concentrations by pGz may be explained on the basis of the multiple effects of NO as a cardioprotective molecule [72–78]. These beneficial effects induced by pGz appear to be mediated by increased production of NO since pretreatment with L-NAME, an inhibitor of nitric oxide synthase blocks the pGz effect on intracellular [Ca^2+^], [Na^+^] and pH. Normalization of Ca^2+^ homeostasis in salvageable cardiomyocytes might also reduce cell death by necrosis and apoptosis, decreasing the infarct size and reducing remodeling [79].

Limitations to the present study must be acknowledged. pGz increases serum prostaglandins and adrenomedullin as well as myocardial nNOS, all which are cardioprotective [6, 8, 10, 14, 80–82]. The aforementioned were not analyzed in the current study. Additionally, we have not directly measured NO levels in this model. Notwithstanding these limitations, our data demonstrate that pGz applied after MI improves myocardial function, and favorably diminishes adverse myocardial remodeling after MI. These data are in agreement with early human data, which showed that pGz after chronic MI, improved exercise capacity, and left ventricular function and increased coronary flow reserve [83, 84].

## Conclusions

The present study demonstrates that a non-pharmacologic, non-invasive intervention which increases pulsatile shear stress to the vascular endothelium, can protect the heart after MI. There are significant clinical applications to the pGz stimulus. In addition to being a preconditioning stimulus, pGz may serve as a simple therapeutic modality to improve myocardial function after MI. pGz is particularly suited for individuals who cannot or will not exercise due to physical or mental limitations. Since, pGz has been shown to be safe in healthy and diseased human individuals and unlike exercise does not require subject cooperation, translation of its use in humans post MI warrants clinical investigation [7, 84, 85].

## Supporting Information

S1 Table Modified Assessment Scoring Criteria and Acute Pain Assessment for rat survival surgery and post-operative care. (PDF)
