# Supplementary material for: Non-Invasive Technology That Improves Cardiac Function after Experimental Myocardial Infarction: Whole Body Periodic Acceleration (pGz)
Source: PLoS One. 2015 Mar 25;10(3):e0121069. doi: 10.1371/journal.pone.0121069 (PMC4373845; doi:10.1371/journal.pone.0121069)
Supplement: S1 Table — (PDF) [file pone.0121069.s001.pdf]

Non-Invasive Technology that Improves Cardiac Function after Experimental Myocardial Infarction: Whole Body Periodic Acceleration (pGz)

Supporting Information

Arkady Uryash [1¶], Jorge Bassuk [1¶†], Paul Kurlansky [2&], Francisco Altamirano [3&],

Jose R. Lopez[3&], Jose A. Adams [1¶*]

[1] Division of Neonatology, Mount Sinai Medical Center, Miami Beach, FL

[2] Columbia Heart Source, Columbia University College of Physicians and Surgeons,

New York, NY

[3] Departments of Molecular Bioscience, School of Veterinary Medicine, University of California Davis, CA

Corresponding author

E-mail: tony-adams@msmc.com

[¶] These authors contributed equally to this work.

[&] These authors also contributed equally to this work.

[†] Deceased

S 1 TABLE

Animal Care Survival Surgery Post Op Assessment & Scoring

			Protocol Number				
Study Date							
WEIGHT							
	Date/Time	Date/Time	Date/Time	Date/Time	Date/Time	Date/Time	Date/Time
Observation							
Inactive							
Walking on Tiptoe							
Hunched posture							
Sunken Eyes							
Ruffled Coat							
Stool[a]							
Handling							
Not Alert							
Not Eating							
Aggressive							
Respirations[b]							
Body Weight(gms)							
% Change							
Body Temp (^{o}C) (if <35°C active warming)							
Overall Condition [c]							
Surgical Site:[d]							
Comment:							
Initials							

Score Details:

[a]Stool: 0=normal 1= loose 2= pools 3=mucus 4= blood

[b]Respirations: R=rapid S=Shallow L=Labored N=Normal

[c] Overall Condition: 0=Normal to 4= emaciated

[d]Surgical Site: 0=clean and normal 2=crusting no pus 3=redness and pus

Acute Pain Assessment in Rats Post Surgery

	Yes	No
Rubbing or Scratching Surgical Site		
Piloerection		
Decreased Grooming		
Arching Back		
Falling or Staggering		
Restlessness or Aggressive		
Increased Respirations		
Reduced Level of Activity		
Porphyrin discharge (eyes or nostrils)		
Vocalization		
